# Spatial-Temporal Differentiation Analysis of Agricultural Land Use Intensity and Its Driving Factors at the County Scale: A Case Study in Hubei Province, China

**DOI:** 10.3390/ijerph17186910

**Published:** 2020-09-21

**Authors:** Li Yu, Zhanqi Wang, Hongwei Zhang, Chao Wei

**Affiliations:** 1 Department of Land Resource Management, School of Public Administration, China University of Geosciences(Wuhan), Wuhan 430074, China; yuli@cug.edu.cn (L.Y.); zhangfocus@cug.edu.cn (H.Z.); 2 School of Politics, Law and Public Administration, Hubei University, Wuhan 430074, China; weichao@cug.edu.cn

**Keywords:** spatial-temporal differentiation, agricultural land use intensity, county scale, Hubei Province

## Abstract

Scientifically characterizing the spatial-temporal distribution characteristics of agricultural land use intensity and analyzing its driving factors are of great significance to the formulation of relevant agricultural land use intensity management policies, the realization of food safety and health, and the achievement of sustainable development goals. Taking Hubei Province as an example, and taking counties as the basic evaluation unit, this paper establishes an agricultural land use intensity evaluation system, explores the spatial autocorrelation of agricultural land use intensity in each county and analyzes the driving factors of agricultural land use intensity. The results show that the agricultural land use intensity in Hubei Province increased as a whole from 2000 to 2016, and the spatial agglomeration about the agricultural land use intensity in Hubei Province experienced a process of continuous growth and a fluctuating decline; the maximum of the Global Moran’s I was 0.430174 (in 2007) and the minimum was 0.148651 (in 2001). In terms of Local Moran’s I, H-H agglomeration units were mainly concentrated in two regions: One comprising the cities of Huanggang, Huangshi and Ezhou, and the other the cities of Xiangyang and Suizhou; the phenomenon is particularly obvious after 2005. On the other hand, factors such as the multiple cropping index (MCI) that reflect farmers’ willingness to engage in agricultural production have a great impact on agricultural land use intensity, the influence of the structure of the industry on agricultural land use intensity varies with the degree of influence of different industries on farmers’ income, and agricultural fiscal expenditure (AFE) has not effectively promoted the intensification of agricultural land use. The present research has important significance for enhancing insights into the sustainable improvement of agricultural land use intensity and for realizing risk control of agricultural land use and development.

## 1. Introduction

Agricultural land use intensity not only has a direct impact on food security, but also affects the realization of rural sustainable development goals [1]. In order to meet the demand for food through agricultural development, China has been increasing its food production capacity by increasing agricultural input for decades, and agricultural land use intensity has changed dramatically. Therefore, it is necessary to characterize the spatial-temporal characteristics of agricultural land use intensity in order to explore the internal law to provide a reference for future agricultural production practice.

Research on agricultural land use intensity has focused on the methods or indicators of characterizing agricultural land use intensity [2], its impact on social-economics development, its impact on the ecological environment [3] and so on. In terms of the methods of characterizing agricultural land use intensity, research showed that: (i) a new system has been constructed using farm accounting network data that include land use, socio-economic factors, local climate, and government subsidies to calculate the unit land cost input [4]; (ii) Polynomial regression models have been applied to detect the spatial distribution of agricultural intensity in France in order to provide an important reference for the implementation of related agricultural policies [5]; and (iii) Some scholars still use indicators of the inputs or outputs to measure agricultural intensification based on traditional agricultural statistics. Indicators of inputs include fertilizers, intercropping levels [6,7], nitrogen input (for arable land and permanent grassland), livestock unit density and pesticide amounts (herbicide, insecticide, and flame-retardant herbicide and insecticide) [8,9], while indicators of the outputs include cereal and animal husbandry products [10], which have only been applied to analyze the characteristics of agricultural land use and their informative significance, rather than focusing on the innovation of characterization methods [11,12,13]. Despite the development of remote sensing science, few scholars have used remote sensing images to characterize agricultural land use intensity dynamically and in real time [14,15,16,17]. On the other hand, the impact of agricultural land use intensity on socio-economic development has been shown to be mainly concentrated around the relationship with population [18], urbanization [19,20], rural transformation [21], food production [22], and food security [23], which have effectively guided the coordinated and sustainable development of intensive agricultural land use [24]. Research on the impact of agricultural land use intensity on the ecological environment has also become widespread in recent decades, mainly focusing on the impact on biodiversity [25,26,27], plant diversity [28,29], the microbial living environment [30,31], landscape structure [32], river health [33], and so on. Some studies have also comprehensively discussed whether the increase in agricultural land use intensity has significantly increased ecological-environmental risks. Only a few scholars have discussed the driving factors of agricultural land use intensity, and related studies considered that these factors included farm characteristics, economic conditions, accessibility, soil conditions, climate conditions, and the increase in nonagricultural job opportunities [34,35].

In general, research on agricultural land use intensity is rich, involving not only the methods or indicators of characterizing agricultural land use intensity, but also the interactions with economic–social development and the ecological environment. However, there are several aspects that need to be further explored: Firstly, the scale of research needs to be expanded; existing research has mainly focused on the national or regional scales, while there is no research on the county level, which is the basic unit of agricultural production in China. Secondly, the influence mechanism and interaction between different factors regarding the willingness of farmers to develop the agricultural production, the structure of economic development and government technology support on the intensive use of agricultural land have not been sufficiently studied, which is clearly not conducive to the formulation of overall land use intensity management policies.

Therefore, this paper takes Hubei Province in China as an example to study the spatial–temporal characteristics of agricultural land use intensity at the county scale, and explores the driving factors of agricultural land use intensity in different units, in the hope of providing a decision-making reference for the formulation of regional agricultural land intensive use policies based on the county areas.

## 2. Study Area and Data

### 2.1. Study Area

**Figure 1:**
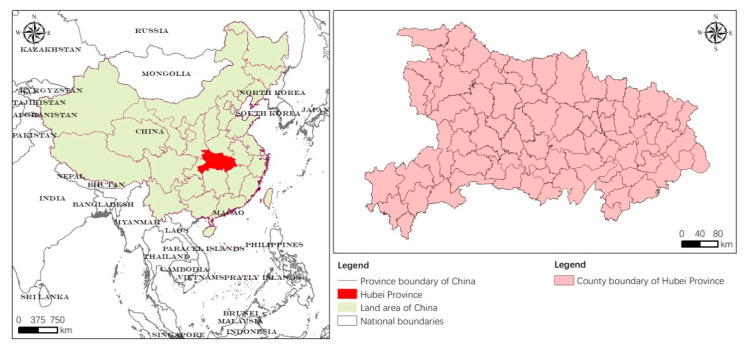
Location of the study area.

Hubei Province is located in central–southern China (Figure 1). It is bordered by six provincial administrative regions, Anhui, Jiangxi, Hunan, Chongqing, Shaanxi and Henan. The province has a total jurisdiction of 103 county administrative districts. Furthermore, it is located in the transition zone of China’s topography: The terrain is diverse, and the topography differs greatly. It is surrounded by mountains on three sides, and the total mountainous area is large and generally shows a trend of high mountains in the northwest and low mountains in the southeast. Industry in Hubei Province developed early, so there is a good industrial and scientific-technological foundation, an overall well-developed rural economy and abundant agricultural labor resources, which have laid a good foundation for improving land use efficiency. The main land use types in Hubei Province are cultivated land, forest land, grassland, water area, construction land and unused land. According to the relevant statistical data, the total area of cultivated land in Hubei Province was 5,235,395 hectares at the end of 2018, of which paddy fields, irrigated land and dry land comprised 2647780.29 hectares, 479410.81 hectares and 2108114.29 hectares, accounting for 50.58%, 9.16% and 40.27% of the total cultivated land area, respectively. The spatial distribution of cultivated land resources in the province is extremely uneven. The plains along the Jianghan and East Hubei plains and the hilly areas of central-northern Hubei are relatively flat with fertile soil and good cultivated land quality, while the cultivated land of the mountainous areas of western Hubei is concentrated in valleys and intermountain basins.

### 2.2. Data Sources

The basic data required for this study included vector and statistical data. Statistical data comprised the agricultural input–output and driving factor data of 103 counties (including municipal districts and county-level cities) in Hubei Province from 2000 to 2016, including the total area of cultivated land, the permanent population, agricultural fertilizer use and total power of agricultural machinery, total agricultural output value, and total grain output, which were taken from the China Regional Economic Statistics Yearbook, the China City Statistical Yearbook, the China Statistical Yearbook and the China Rural Statistical Yearbook. The vector data were taken from the National Basic Geographic Information Bureau and Hubei Provincial Department of Natural Resources. It should be noted that because of changes in administrative divisions and data availability, some data were deleted and merged.

## 3. Methodology

### 3.1. Agricultural Land Use Intensity Indices (In and Out)

This paper posits that evaluating the level of agricultural land use intensity provides a comprehensive measurement of the input and output of cultivated land; as such, it should not only measure the input intensity, but also reflect the utilization efficiency of cultivated land. Therefore, according to the connotation and objectives of agricultural land use intensity, combined with the current characteristics of cultivated land use in Hubei Province, the indicators were constructed from the inputs and outputs on the basis of previous research results, i.e., Chemical fertilizers per unit of cultivated land, farming mechanical power per unit of cultivated land, agricultural film per unit of cultivated land, etc., which directly reflect the input intensity of farmland and the average output. The specific system of the indicators is as follows (Table 1):

### 3.2. Assessment of Agricultural Land Use Intensity

#### 3.2.1. Data Standardization

Different units are applied to different indicators, and the differences in the values of the indicators are extremely large, resulting in incomparability. In order to solve this problem, the data must be standardized. The standard deviation standardization method, the extreme value standardization method, the sum standardization method and the range standardization method are commonly applied. This paper adopted the range standardization method to process the dataset of each indicator. The specific process was as follows:

The positively correlated indicators were standardized using the following formula:

(1) $$C_{ij}=\left(X_{ij}-X_{jmin}\right)/\left(X_{jmax}-X_{jmin}\right)$$

The negatively correlated indicators were standardized as follows:

(2) $$C_{ij}=\left(X_{jmax}-X_{ij}\right)/\left(X_{jmax}-X_{jmin}\right)$$

where $C_{ij}$ is the value after normalization, $X_{ij}$ is the statistical value of each indicator of each evaluation cell, *j* is a different evaluation indicator, *I* is a different evaluation unit corresponding to indicator *j*, $X_{jmin}$ refers to the minimum value of indicator *j* and $X_{jmax}$ is the maximum value in the indicators of indicator *j*.

#### 3.2.2. Comprehensive Evaluation Method

The determination of indicator weight is based on the premise of comprehensive indicator calculation. This paper used a combination of the entropy weight and the analytic hierarchy process methods to determine the indicator weight. The entropy weight method is for objective weighing, while the analytic hierarchy process method is for subjective weighing. The calculation formula of the final weight and the calculation process of the comprehensive score are as follows:

Firstly, the average of the weights of the two weighing methods was calculated; the calculation formula was as follows:

(3) $$\alpha_{j}=\frac{\beta_{j}+\gamma_{j}}{2}\left(1\leq j\leq m\right)$$

where $\beta_{j}$ is the weight value of the *j* th indicator obtained by the analytic hierarchy process method, $\gamma_{j}$ is the weight value of the *j* th indicator obtained by the entropy weight method and $\alpha_{j}$ is the combined weight value of the *j* th indicator.

The variable $\alpha_{j}$ was normalized to obtain the final combined weight value of each indicator:

(4) $$\delta_{j}=\frac{\alpha_{j}}{\sum_{j=1}^{m}\alpha_{j}}\left(1\leq j\leq m\right)$$

Secondly, the weights of the indicators were determined by the weight determination method, and the comprehensive score of each evaluation unit was determined using the following formula:

(5) $$QI_{i}=\left(\sum_{j=1}^{n}q_{ij}\delta_{ij}\right)\times 100$$

where $QI_{i}$ is the comprehensive score of the agricultural land use intensity of the *I* th evaluation unit, $\delta_{ij}$ is the weight value of indicator *j*, and the statistical value corresponding to indicator *j* of the *I* th evaluation unit after $q_{ij}$ was standardized and normalized.

### 3.3. Spatial-Temporal Differentiation Analysis

#### 3.3.1. Descriptive Statistical Analysis

Descriptive statistics is a basic method that is used to summarize and express an overall condition and the influence between different indicators. Through simple statistical values, we can clearly grasp the overall characteristics of the evaluation results, and fully understand and identify the concentrated or discrete nature of data. Therefore, this study constructed a descriptive statistical variable system that included six statistical values, namely, the arithmetic mean (hereinafter referred to as “average”), median, maximum, minimum, standard deviation and coefficient of variation, to grasp the basic situation of agricultural land use intensity.

#### 3.3.2. Spatial Autocorrelation

Spatial autocorrelation refers to the mutual restraint, interdependence, interaction and mutual influence in the geographical space between the objects and phenomena of different units, which are the inherent space economy of objects and phenomena, and are also essential attributes of geospatial phenomena and spatial processes. When a high value of the characteristic variable of the adjacent unit shows a spatial tendency to agglomerate, it displays a positive spatial autocorrelation. In contrast, when the value of the characteristic variable of the adjacent unit is opposite to the value of the variable of the local unit, it displays a negative spatial autocorrelation.

Current research on spatial autocorrelation measurements includes Global spatial autocorrelation and Local spatial autocorrelation. Global spatial autocorrelation is the examination of the average influence on and the attributes of a particular spatial degree, while local spatial autocorrelation is mainly used to test whether there are similar or different observations in the local area. The indicators applied in the current study were the Global Moran’s I and Local Moran’s I (Anselin Local Moran’s I). The specific calculation process is as follows:

(i) Global Moran’s I

Global Moran’s I is used to evaluate whether the expressed mode is a clustering mode, a discrete mode or a random mode. The value of Moran’s I can be regarded as the correlation coefficient between the observed value and its spatial lag. The specific formula is as follows:

(6) $$I=\frac{n}{S_{0}}\frac{\sum_{i=1}^{n}\sum_{j=1}^{n}w_{i,j}z_{i}z_{j}}{\sum_{i=1}^{n}z_{i}^{2}}$$

In the results, the value of Moran’s I is generally between −1 and 1. If the value of Moran’s I is positive, the positive spatial correlation of this element is strong (in particular, a high value is adjacent to a high value, and a low value is adjacent to a low value), but if the value of Moran’s I is negative, the element has a strong discrete trend and the correlation is not very pronounced (in particular, a high value is adjacent to a low value, and a low value is adjacent to a high value). If Moran’s I is close to 0, it means that the attributes are randomly distributed (or there is no spatial autocorrelation).

(ii) Anselin Local Moran’s I

Anselin Local Moran’s I is an indicator that was proposed by Anselin in 1995 to test the spatial autocorrelation between a local unit and its neighboring units, which can effectively solve the problem of the inability of Global spatial autocorrelation to accurately represent the aggregation or the specific geospatial location of the spatial autocorrelation between a local unit and its neighboring units. The specific calculation formula is as follows:

(7) $$I_{i}=\frac{x_{i}-\bar{X}}{S_{i}^{2}}\sum_{j=1,j\neq i}^{n}w_{i,j}\left(x_{j}-\bar{X}\right)$$

A positive $I_{i}$ indicates that the value is high and surrounded by high values, or it is low and surrounded by low values; a negative $I_{i}$ indicates that the value is low but surrounded by high values, or that the value is high and surrounded by low values. In this paper, Local indicators of spatial association (LISA) figures of Local Moran’s I on agricultural land use intensity were drawn.

#### 3.3.3. Spatial-Temporal Transition

The spatial-temporal transition of spatial autocorrelation was proposed by Rey according to the transfer of the quadrant to which each evaluation unit belongs in the Moran scatter diagram in different periods [36], which can reflect the stability of the spatial autocorrelation of the research unit. The types of spatial–temporal transitions include the following: Type A, which is the transition of the region’s agricultural land use intensity, and no transition occurs in the adjacent units (low-low (L-L)↔high-low (H-L), high–high (H-H)↔low-high (L-H)); Type B, which is the same level as its agricultural land use intensity, and transition occurs in the adjacent units (H-H↔H-L, L-L↔L-H); Type C, which means that the level of agricultural land use intensity has changed, and the level of the adjacent units has also changed (H-H↔L-L, L-H↔H-L); Type D, which is the unit itself, and there is no transition in the adjacent units. Its spatial stability can be defined as:

(8) $$S_{t}=\frac{N_{d,t}}{n}$$

where $S_{t}$ represents the spatial stability in the time range of *t*, $N_{d,t}$ represents the number of regions where type D transitions occur in the time range of *t* and *n* is the total number of all types of transitions that occur; the larger the value, the stronger the spatial stability.

### 3.4. Driving Factor Analysis Model

#### 3.4.1. Indicators of Driving Factors

This paper used the multiple cropping index (MCI) and the irrigation index (II) to represent the willingness of farmers to develop agricultural production, as well as the per capita output value of primary industry (PCOVPI), per capita output value of secondary industry (PCOVSI), and per capita output value of tertiary industry (PCOVTI) to represent the differences in the structure of economic development and agricultural fiscal expenditure (AFE) as indicators of government technology support, and to explore the core factor leading to changes in agricultural land use intensity (Table 2).

#### 3.4.2. Geographical Detectors

Factor detection: The mechanism of the influence of agricultural land use intensity was analyzed in counties using a geographical detector. The geographical detector model is as follows [37]:

(9) $$P_{D,U}=1-\frac{1}{nu_{U}^{2}}\sum_{i=1}^{m}n_{D,i}\sigma_{U_{D,i}}^{2}$$

where $P_{D,U}$ is the detection factor of the driving factors on agricultural land use intensity, $n_{D,i}$ is the number of samples of the secondary region, *n* is the number of samples in the whole area, *m* is the number in secondary regions, $u_{U}^{2}$ represents the variance of the degree of dynamicity of agricultural land use intensity changes and $\sigma_{U_{D,i}}^{2}$ represents the variance of secondary regions. Formally, $\sigma_{U_{D,i}}^{2}\neq 0$, and the range of $P_{D,U}$ is [0,1]. When $P_{D,U}$ = 0, it indicates that the agricultural land use intensity at the county level is randomly distributed; the higher the value of $P_{D,U}$, the stronger the influence of the driving factor on the degree of agricultural land use intensity.

Interaction detection: This was used to identify the interaction between different risk factors (Xs) and to assess whether the factors X1 and X2 work together to increase or decrease the explanatory power on the dependent variable Y, or whether the impacts of these factors on Y are independent of each other. The first step of the evaluation method is to calculate the q values of the two factors, X1 and X2, to Y, q(X1) and q(X2). Then the q value is calculated of their interaction (the new layer formed by the tangency of the two layers of the superimposed variables X1 and X2): q(X1∩X2), and q(X1), q(X2), and q(X1∩X2) are compared. The relationship between the two factors can be divided into the categories outlined in Table 3.

## 4. Results

### 4.1. Descriptive Statistical Analysis of Agricultural Land Use Intensity

The results showed that the average value (mean) of agricultural land use intensity in Hubei Province from 2000 to 2016 fluctuated and increased. But after 2008, although the overall level was still high, there was a downward trend. Similarly, the median of the agricultural land use intensity in Hubei Province basically showed the same change law as the mean, which can be further verified by the observed decrease in the maximum and minimum of the values. At the same time, the standard deviation and variation coefficient of the agricultural land use intensity also increased over that decade, and there was a decline after 2008, albeit a smaller one. The results are shown in Table 4.

### 4.2. Analysis of the Spatial Autocorrelation

From the *p* -Value, Var and Z-Value, it can be seen that agricultural land use intensity by county in Hubei Province passed the 1% significance test from 2008 to 2017, and presented a pattern of spatial aggregation distribution between agricultural land use intensity in various counties. On the other hand, the values of Global Moran’s I were positive in each year, indicating that the county-level agricultural land use intensity had positive spatial autocorrelation. However, the value of Global Moran’s I showed a trend of increasing volatility from 2000 to 2007, and a trend of decreasing volatility after 2008, which indicated that the agglomeration of the corresponding counties with similar agricultural land use intensity had a tendency to decrease. Within this period, the year with the lowest Global Moran’s I was 2001, indicating that the spatial autocorrelation of agricultural land use intensity was weak. In contrast, the highest Global Moran’s I was in 2007, indicating that Hubei Province experienced the highest spatial autocorrelation of agricultural land use intensity in this year (Table 5).

**Figure 2:**
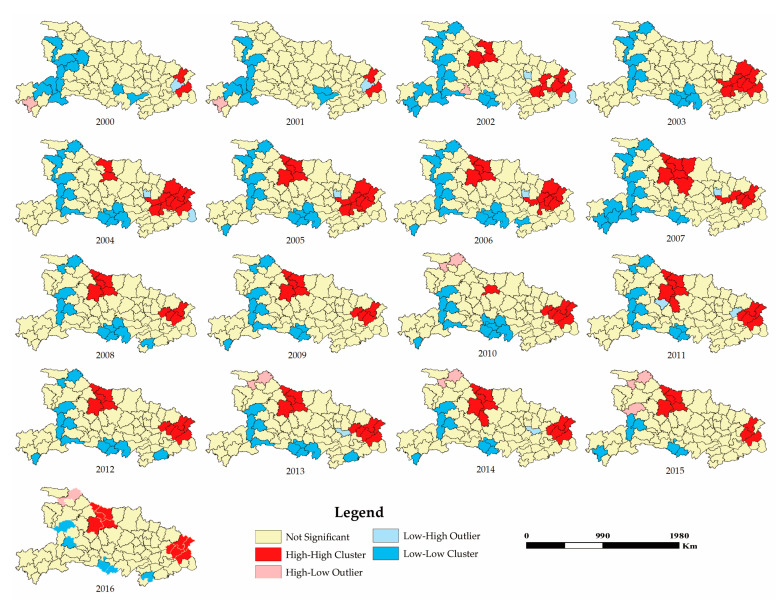
LISA figures on agricultural land use intensity in Hubei Province from 2000 to 2016.

Since Global Moran’s I is a regional overall measurement index, it cannot describe the spatial position of the strength of the spatial autocorrelation of agricultural land use intensity. Therefore, on the basis of its calculation, LISA figures of Local Moran’s I on agricultural land use intensity were drawn for each county in Hubei Province from 2000 to 2016. The results are shown in Figure 2. On the whole, the H-H agglomeration units were concentrated in two regions: One comprising the cities of Huanggang, Huangshi and Ezhou, and the other comprising the cities of Xiangyang and Suizhou; and this phenomenon was particularly noticeable after 2005. Also, the regions of the H-H agglomeration units maintained a stable aggregation state after 2005. On the other hand, the L-L agglomeration units presented an L-shaped distribution from the cities of Yichang, Shiyan and the forest area of Shennongjia to the city of Jingzhou, as well as a decreasing trend over time. The other types of agglomeration units showed sporadic distribution.

### 4.3. Spatial-Temporal Transition Analysis

On the basis of the principle of spatial-temporal transition and considering a time interval of 17 years, the spatial–temporal transition law of agricultural land use intensity in each county of Hubei Province was analyzed. The blue shaded numbers in Table 6 indicate how many times the transfer happened in the year. The results show that there were many D-type unit transitions during the 17 years, which generally occupied about 80% of all evaluation units; fewer evaluation units showed types A, B, and C transitions. In contrast to the principle of spatial–temporal transition analysis, the actual analysis involves the transition from nonsignificant units to salient units and from salient units to nonsignificant units. If they were not considered, the spatial stability of agricultural land use intensity was stronger, and there was almost no change (Table 6).

### 4.4. Driving Factor Analysis

The decisive results of the driving factors of agricultural land use intensity according to the geographical detectors are shown in Table 7; the q statistics represent the size of the driving power($P_{D,U}$) and *p*-Value represents the result of the significance test. From the detection results, the MCI, II, PCOVPI, PCOVSI, PCOVTI, and AFE were shown to have a significant positive impact on the intensity of agricultural land use development, and the effect of the MCI on agricultural land use intensity was more pronounced, but its influence showed a downward fluctuation trend which was more noticeable after 2008. The impact of II was relatively small, but it grew rapidly from 2000 to 2007, after which it showed a downward trend. From the perspective of PCOVPI, PCOVSI and PCOVTI, a fluctuating growth trend can be observed, in which the PCOVSI had the fastest-growing impact on agricultural land use intensity. In contrast to the other influencing factors, the impact of AFE on agricultural land use intensity always showed a downward trend in terms of volatility, and its impact on the intensity of agricultural land use was minimal.

After interactively detecting the six driving factors from 2000 to 2016, 15 effective impact factor pairs were obtained, the specific results of which are shown in Table 8. On the whole, the results of the interaction detection showed that the influence of various driving factors on the intensity of agricultural land use was interactive. The interaction of MCI and II, MCI and PCOVTI, MCI and AFE, II and PCOVPI, II and PCOVTI, II and AFE, PCOVPI and PCOVSI, PCOVPI and PCOVTI, PCOVPI and AFE, PCOVSI and PCOVTI, PCOVSI and AFE, PCOVTI and AFE showed the basic characteristics of nonlinear enhancement. The interaction of MCI and PCOVPI showed the basic characteristics of two-factor enhancement, but the interaction of II and PCOVSI showed nonlinearity enhanced basic features. From the perspective of time series, the degree of interaction showed a trend of increasing volatility before 2012, but a trend of declining volatility after 2012 which was more pronounced.

## 5. Discussion

Compared to the research of other scholars on the impact of agricultural land use intensity on surface water quality, biodiversity, etc. [38], on the basis of the county as the basic agricultural production unit, this paper only discussed the geographical spatial correlation of agricultural land use intensity and its driving factors. This part discusses important insights and the significance of this spatial correlation for agricultural land use development intensity management based on the aforementioned geospatial correlation research.

### 5.1. Agglomeration Effect of Agricultural Land Use Intensity

Judging from the change in the Global Moran’s I, the values of Global Moran’s I had experienced a trend of increasing and decreasing volatility, which showed that the spatial agglomeration of agricultural land use intensity in Hubei Province gradually decreased over the time, and the reason may be that the spatial agglomeration of agricultural land use intensity was decreasingly affected by the natural endowment conditions of agricultural land at this stage, and that the development of modern agriculture may have been more concerned with the needs of residents, but previous research focused on the impact of the natural endowment of agricultural land on agricultural land use intensity [39]. Considering the results of the local spatial autocorrelation analysis, the units with greater agricultural land use intensity were agglomerated in the cities of Xiangyang or Wuhan where are flat. In the early stage of rapid economic social development, the agglomerations were increasingly noticeable, but with the passage of time, local spatial agglomerations gradually became smaller, the trend is not pronounced, and they showed a stable state.

Research had shown that the regionally of agricultural land use intensity was greatly affected by natural endowment conditions [40], which determined the initial agglomeration pattern of agricultural land use intensity, but the rapid development of the economy and society changed the regional of agricultural land use intensity. Therefore, the intensity of agricultural land use was affected by multiple factors and different factors, and the time nodes were not the same.

At the same time, an important finding in this paper was that in regions with better ecology, such as the city of Shiyan and the forest area of Shennongjia in Hubei Province, the intensity of agricultural land use was not very high. Therefore, in the process of continuing the research on the relationship between agricultural land use intensity, ecological environment protection, and biodiversity, it is necessary to continuously improve relevant knowledge.

### 5.2. Driving Factors of the Agglomeration Effect

The spatial-temporal differentiation of agricultural land use intensity in the counties of Hubei Province from 2000 to 2016 was significantly affected by the willingness of agricultural farmers to develop their lands, differences in economic structure and governmental support of science and technology. Among them, the MCI had the strongest ability to determine farmers’ willingness to develop, showing that the intensity of agricultural land use is still closely related to this parameter. On the other hand, primary, secondary and tertiary industries became the main determinants in different years. An underlying reason for this may be that part of farmers’ income comes from agricultural production, but what is more important is the wage income generated by their work, and the industry that they are engaged in is constantly changing with changes in their income level. The impact of governmental agricultural finance or scientific-technological support on the intensity of agricultural land use was not as great as we imagined; this may rather have an important relationship with the country’s basic household contract responsibility system. Therefore, if the government wants to increase the level of agricultural development through financial or scientific-technological investment, it is an important prerequisite to actively realize the scale management and land circulation of agricultural land.

It should be pointed out that the results of this research do not conflict with those of other scholars regarding the driving factors of agricultural land use intensity. Agricultural land use intensity is comprehensively affected by factors such as natural endowment conditions and economic-social comprehensive levels [41,42]; this paper showed that the former determines the initial regional intensity of agricultural land use, while subsequent regional fluctuations in agricultural land use intensity are more affected by factors such as differences in internal economic structure, farmers’ wishes with regard to social factors and governmental policy support.

### 5.3. Agricultural Land Use Intensity Management Policy Formulation

Through the analysis of spatial–temporal differentiation characteristics, it was found that agricultural land use intensity is characterized by high regional on the basis of the basic agricultural production unit of each county. In terms of its driving factors, the increase in intensity was closely related to the willingness of farmers to carry out agricultural activities and the nonagricultural income provided to farmers by other types of industrial development. Therefore, in future agricultural land use policy formulation processes, we should start from the following aspects: First, increasing the added value of the agricultural industry will increase the level of enthusiasm of farmers; Second, continuing to encourage large-scale operations will also promote the improvement of agricultural land use intensity to a certain extent; Third, the agglomeration characteristics of agricultural land use intensity indicate that the formulation of a rational agricultural land use intensity management strategy should fully consider regional variations; And fourth, the units with greatest intensity of agricultural land use were near the Wuhan City Circle. These units should be vigilantly monitored to control the impact on people’s health of such intense activity.

## 6. Conclusions

This research shows that agricultural land use development has obvious regional characteristics, and although its degree of agglomeration also showed a certain fluctuation in correlation with economic and social fluctuations, it showed strong stability over time. Therefore, measurements that need to be taken on improving land use intensity should not start with a single unit, which should consider the entirety of the region, and that is more conducive to agricultural sustainability and stability. For units that need to reduce the intensity of agricultural land use, it is also necessary to consider the intensity of agricultural land use in adjacent units so as to achieve a regional health, production health and ecological balance.

The identification of the driving factors of agricultural land use intensity under the influence of geographical factors showed that the MCI of the representative index of human cultivation intention has a significant impact on agricultural land use intensity. In the process of enhancing agricultural land use intensity, continuing to promote the reform measures of land management in the new economic-social development stage such as family farms and rural planting cooperatives effectively promotes land circulation and achieves large-scale operations which can reduce the dependence of agricultural production efficiency on farmers’ wishes and ultimately promote the development of modern agriculture.

Counties are the basic agricultural production unit in China, the characteristics presented in the different stages of their development are quite different, and the level of agricultural land intensive use is affected by the interaction of many factors. Therefore, in formulating policies for agricultural land use management, the comprehensiveness and current status of policy formulation should be strengthened. This will be of great significance to increasing or decreasing the intensity of agricultural land use in China.

## Figures and Tables

**Table 1:** The Indicators of Agricultural Land Use Intensity.

Indices	Indicators	Definition	Remarks
Input	A1	Consumption of chemical fertilizers per unit of cultivated land	Represents the capital component of production input
	A2	Farming mechanical power per unit of cultivated land	Represents the capital component of production input
	A3	Consumption of agricultural film per unit of cultivated land	Represents the capital component of production input
	A4	Consumption of agricultural diesel per unit of cultivated land	Represents the capital component of production input
Output	A5	Agricultural electricity consumption per unit of cultivated land	Reflect the situation of agricultural production
	A6	Gross Agricultural Output Value per unit of cultivated land	Reflect the situation of agricultural production

**Table 2:** The Driving Factors of Agricultural Land Use Intensity.

Variables	Definition	Sources of Data
Multiple cropping index (MCI)	Ratio of total sown area of crops to cultivated area	The China Statistical Yearbook (county-level) and the Statistical Yearbook of Hubei Province
Irrigation index (II)	Ratio of irrigated area to cultivated area	The China Statistical Yearbook (county-level) and the Statistical Yearbook of Hubei Province
Per capita output value of the primary industry (PCOVPI)	Ratio of the output value of primary industry to the permanent population	The China Statistical Yearbook (county-level) and the Statistical Yearbook of Hubei Province
Per capita output value of the secondary industry (PCOVSI)	Ratio of the output value of secondary industry to the permanent population	The China Statistical Yearbook (county-level) and the Statistical Yearbook of Hubei Province
Per capita output value of the tertiary industry (PCOVTI)	Ratio of the tertiary industry output value to the permanent population	The China Statistical Yearbook (county-level) and the Statistical Yearbook of Hubei Province
Agricultural fiscal expenditure (AFE)	Agricultural fiscal expenditure	The China Statistical Yearbook (county-level) and the Statistical Yearbook of Hubei Province

**Table 3:** Types of Interactions between Driving Factors.

Judgment Basis	Interaction
q(X1∩X2) < Min(q(X1),q(X2))	Nonlinear attenuation
Min(q(X1),q(X2)) < q(X1∩X2)<Max(q(X1),q(X2))	Single-factor nonlinear attenuation
q(X1∩X2) > Max(q(X1),q(X2))	Two-factor enhancement
q(X1∩X2) = q(X1) + q(X2)	Independent
q(X1∩X2) > q(X1) + q(X2)	Nonlinear enhancement

**Table 4:** Descriptive Statistical Analysis of Agricultural Land Use Intensity in Hubei Province from 2000 to 2016.

	Year	2000	2001	2002	2003	2004	2005	2006	2007	
Variables										
**Mean**		**1.913**	**1.725**	**1.479**	**1.446**	**2.148**	**2.342**	**2.305**	**2.062**	
Median		1.876	1.629	1.394	1.432	2.148	2.370	2.293	2.002	
Maximum		4.729	4.730	4.698	4.623	4.433	4.241	4.193	4.462	
Minimum		0.591	0.592	0.516	0.571	0.815	1.010	0.825	0.354	
Standard Deviation		0.529	0.534	0.641	0.626	0.720	0.747	0.797	0.901	
Variation Coefficient		0.277	0.310	0.433	0.433	0.335	0.319	0.346	0.437	
	**Year**	**2008**	**2009**	**2010**	**2011**	**2012**	**2013**	**2014**	**2015**	**2016**
**Variables**										
Mean		2.293	2.233	2.026	2.063	2.334	2.097	2.038	1.953	2.004
Median		2.265	2.264	2.026	2.081	2.396	2.122	2.107	1.981	1.982
Maximum		4.427	4.419	4.539	4.072	4.162	3.911	3.628	3.472	3.695
Minimum		0.697	0.731	0.658	0.695	0.000	0.716	0.703	0.646	0.812
Standard Deviation		0.817	0.768	0.680	0.704	0.737	0.710	0.650	0.636	0.647
Variation Coefficient		0.356	0.344	0.335	0.341	0.316	0.339	0.319	0.326	0.323

**Table 5:** Global Moran’s I of Agricultural Land Use Intensity in Hubei Province from 2000 to 2016.

Year	Moran’s I	Var	Z-Value	*p*-Value
2000	0.230089	0.004130	3.774936	0.000160
2001	0.148651	0.003954	2.562859	0.010381
2002	0.374362	0.004303	5.897530	0.000000
2003	0.328729	0.004271	5.221184	0.000000
2004	0.384508	0.004667	5.811456	0.000000
2005	0.391518	0.004696	5.895653	0.000000
2006	0.396168	0.004697	5.962794	0.000000
2007	0.430174	0.004681	6.469888	0.000000
2008	0.332978	0.004661	5.060546	0.000000
2009	0.292806	0.004652	4.476065	0.000008
2010	0.313902	0.004585	4.820359	0.000001
2011	0.332591	0.004670	5.049709	0.000000
2012	0.300246	0.004674	4.574698	0.000005
2013	0.293114	0.004688	4.463429	0.000008
2014	0.286077	0.004695	4.357521	0.000013
2015	0.241891	0.004696	3.712302	0.000205
2016	0.241900	0.004685	3.716784	0.000202

**Table 6:** Transfer path of spatial agglomeration on agricultural land use intensity in Hubei Province from 2000 to 2016.

Types	2000-2001	2001-2002	2002-2003	2003-2004	2004-2005	2005-2006	2006-2007	2007-2008	2008-2009	2009-2010	2010-2011	2011-2012	2012-2013	2013-2014	2014-2015	2015-2016
HH→HL	——	——	——	——	——	——	——	——	——	——	——	——	——	——	——	——
HH→LL	——	——	——	——	——	——	——	——	——	——	——	——	——	——	——	——
HH→LH	——	——	——	——	——	——	——	——	——	——	1	——	——	——	——	——
HH→NS	——	——	3	——	2	2	2	4	——	4	——	1	——	1	3	——
HL→HH	——	——	——	——	——	——	——	——	——	——	——	——	——	——	——	——
HL→LL	——	1	——	——	——	——	——	——	——	——	1	——	——	——	——	——
HL→LH	——	——	——	——	——	——	——	——	——	——	——	——	——	——	——	——
HL→NS	——	——	1	——	——	——	——	——	——	——	——	——	——	——	——	——
LL→HH	——	1	——	——	——	——	——	——	——	——	——	——	——	——	——	——
LL→HL	——	——	——	——	——	——	——	——	——	——	——	——	1	——	1	——
LL→LH	——	——	——	——	——	——	——	——	——	1	——	——	——	——	——	——
LL→NS	3	3	5	——	1	——	4	7	3	——	4	——	1	3	2	2
LH→HH	——	——	——	——	——	——	——	——	——	——	——	1	——	——	——	——
LH→HL	——	——	——	——	——	——	——	——	——	——	——	——	——	——	——	——
LH→LL	——	——	——	——	——	——	——	——	——	——	——	——	——	——	——	——
LH→NS	——	——	2	——	1	——	——	1	——	——	——	1	——	——	1	——
NS→HH	——	6	3	3	4	——	1	——	——	2	5	——	——	1	——	2
NS→HL	——	1	——	——	——	——	——	——	——	——	——	——	——	——	——	——
NS→LL	1	7	2	1	1	2	4	4	2	3	——	3	——	1	1	1
NS→LH	——	2	——	2	——	——	——	——	——	——	1	——	1	——	——	——

* NS: Not Significant.

**Table 7:** Results of the Effects of Driving Factors on Agricultural Land Use Intensity.

Year	q Statistic						*p*-Value						Effect Direction					
	MCI	II	PCOVPI	PCOVSI	PCOVTI	AFE	MCI	II	PCOVPI	PCOVSI	PCOVTI	AFE	MCI	II	PCOVPI	PCOVSI	PCOVTI	AFE
2000	0.4518	0.1454	0.0875	0.1499	0.0637	0.0518	0.7268	0.1909	0.8799	0.9936	0.9987	0.9976	+	+	+	+	+	+
2001	0.4942	0.0995	0.2449	0.2258	0.0537	0.0683	0.5547	0.4658	0.4532	0.9596	0.9997	0.9999	+	+	+	+	+	+
2002	0.7536	0.4527	0.1553	0.1413	0.0710	0.0579	0.9985	0.4532	0.2733	0.8471	0.9986	0.9795	+	+	+	+	+	+
2003	0.7582	0.4354	0.1205	0.1510	0.1245	0.0508	0.9876	0.4406	0.4089	0.7326	0.9578	0.9900	+	+	+	+	+	+
2004	0.6540	0.3871	0.1026	0.0911	0.1010	0.0467	0.9767	0.4279	0.9300	0.9984	0.9939	0.9871	+	+	+	+	+	+
2005	0.5944	0.3832	0.0672	0.1124	0.1068	0.0324	0.9658	0.4153	0.9726	0.9999	0.9987	0.9978	+	+	+	+	+	+
2006	0.5264	0.3449	0.1141	0.1353	0.1656	0.1199	0.9549	0.4026	0.8539	1.0000	0.9889	0.8143	+	+	+	+	+	+
2007	0.5638	0.3887	0.0849	0.1334	0.1406	0.0984	0.9440	0.3900	0.8904	0.9993	0.9269	0.8612	+	+	+	+	+	+
2008	0.5463	0.2661	0.0558	0.1818	0.1615	0.0693	0.9331	0.0030	0.9212	0.9744	0.9143	0.9512	+	+	+	+	+	+
2009	0.5159	0.2513	0.0539	0.1952	0.1230	0.0656	0.9222	0.0181	0.9182	0.9231	0.9792	0.9770	+	+	+	+	+	+
2010	0.5321	0.2791	0.0783	0.1257	0.1527	0.0261	0.9113	0.0120	0.9737	0.9913	0.9977	0.9985	+	+	+	+	+	+
2011	0.4280	0.2971	0.1160	0.2089	0.1214	0.0380	0.9004	0.0361	0.9753	0.8630	0.9184	0.9995	+	+	+	+	+	+
2012	0.9819	0.9737	0.0159	0.0594	0.0305	0.0840	0.8895	0.0000	0.9502	0.5336	0.8271	0.3646	+	+	+	+	+	+
2013	0.4855	0.2045	0.0666	0.1903	0.2094	0.0533	0.8786	0.2197	0.9874	0.6419	0.4747	0.9978	+	+	+	+	+	+
2014	0.5027	0.1249	0.1318	0.1908	0.2127	0.0745	0.8677	0.8631	0.8865	0.6856	0.6217	0.9511	+	+	+	+	+	+
2015	0.4059	0.1918	0.2893	0.2427	0.0944	0.0996	0.8568	0.0846	0.1259	0.4804	0.9995	0.9553	+	+	+	+	+	+
2016	0.4331	0.1404	0.1390	0.2319	0.0833	0.0211	0.8459	0.3448	0.9606	0.5183	0.9994	0.9995	+	+	+	+	+	+

“+” represents positive drive.

**Table 8:** Interaction results of different factors on the agriculture land-use intensity in Hubei Province from 2000 to 2016.

*	FZ2000	GG2000	EC2000	SC2000	YC2000	ZC2000	*	FZ2001	GG2001	EC2001	SC2001	YC2001	ZC2001	*	FZ2002	GG2002	EC2002	SC2002	YC2002	ZC2002
FZ2000	0.452						FZ2001	0.494						FZ2002	0.754					
GG2000	0.707	0.145					GG2001	0.678	0.099					GG2002	0.826	0.453				
EC2000	0.602	0.344	0.150				EC2001	0.738	0.399	0.226				EC2002	0.868	0.613	0.141			
SC2000	0.628	0.439	0.286	0.064			SC2001	0.737	0.367	0.276	0.054			SC2002	0.856	0.594	0.202	0.071		
YC2000	0.573	0.406	0.316	0.248	0.087		YC2001	0.782	0.513	0.606	0.742	0.245		YC2002	0.810	0.609	0.503	0.394	0.155	
ZC2000	0.646	0.328	0.289	0.329	0.260	0.052	ZC2001	0.741	0.364	0.324	0.250	0.449	0.068	ZC2002	0.820	0.556	0.231	0.240	0.398	0.058
*	FZ2003	GG2003	EC2003	SC2003	YC2003	ZC2003	*	FZ2004	GG2004	EC2004	SC2004	YC2004	ZC2004	*	FZ2005	GG2005	EC2005	SC2005	YC2005	ZC2005
FZ2003	0.758						FZ2004	0.654						FZ2005	0.594					
GG2003	0.848	0.435					GG2004	0.798	0.387					GG2005	0.746	0.383				
EC2003	0.836	0.575	0.151				EC2004	0.790	0.533	0.091				EC2005	0.667	0.514	0.112			
SC2003	0.861	0.609	0.254	0.125			SC2004	0.812	0.567	0.197	0.101			SC2005	0.660	0.534	0.160	0.107		
YC2003	0.833	0.604	0.405	0.515	0.120		YC2004	0.719	0.583	0.259	0.285	0.103		YC2005	0.696	0.624	0.205	0.190	0.067	
ZC2003	0.802	0.566	0.206	0.229	0.318	0.051	ZC2004	0.759	0.586	0.215	0.271	0.350	0.047	ZC2005	0.688	0.617	0.167	0.188	0.253	0.032
*	FZ2006	GG2006	EC2006	SC2006	YC2006	ZC2006	*	FZ2007	GG2007	EC2007	SC2007	YC2007	ZC2007	*	FZ2008	GG2008	EC2008	SC2008	YC2008	ZC2008
FZ2006	0.526						FZ2007	0.564						FZ2008	0.546					
GG2006	0.712	0.345					GG2007	0.774	0.389					GG2008	0.733	0.266				
EC2006	0.661	0.530	0.135				EC2007	0.725	0.568	0.133				EC2008	0.704	0.475	0.182			
SC2006	0.678	0.573	0.236	0.166			SC2007	0.733	0.582	0.184	0.141			SC2008	0.676	0.532	0.253	0.162		
YC2006	0.621	0.579	0.280	0.303	0.114		YC2007	0.677	0.549	0.300	0.314	0.085		YC2008	0.599	0.583	0.411	0.431	0.056	
ZC2006	0.666	0.571	0.179	0.212	0.334	0.120	ZC2007	0.730	0.638	0.232	0.246	0.346	0.098	ZC2008	0.716	0.385	0.372	0.253	0.306	0.069
*	FZ2009	GG2009	EC2009	SC2009	YC2009	ZC2009	*	FZ2010	GG2010	EC2010	SC2010	YC2010	ZC2010	*	FZ2011	GG2011	EC2011	SC2011	YC2011	ZC2011
FZ2009	0.516						FZ2010	0.532						FZ2011	0.428					
GG2009	0.694	0.251					GG2010	0.771	0.279					GG2011	0.743	0.297				
EC2009	0.667	0.488	0.195				EC2010	0.725	0.464	0.126				EC2011	0.730	0.489	0.209			
SC2009	0.645	0.517	0.276	0.123			SC2010	0.660	0.446	0.268	0.153			SC2011	0.707	0.486	0.263	0.121		
YC2009	0.595	0.469	0.482	0.302	0.054		YC2010	0.759	0.569	0.406	0.412	0.078		YC2011	0.627	0.495	0.422	0.349	0.116	
ZC2009	0.590	0.441	0.328	0.238	0.320	0.066	ZC2010	0.670	0.392	0.306	0.244	0.361	0.026	ZC2011	0.612	0.439	0.340	0.274	0.357	0.038
*	FZ2012	GG2012	EC2012	SC2012	YC2012	ZC2012	*	FZ2013	GG2013	EC2013	SC2013	YC2013	ZC2013	*	FZ2014	GG2014	EC2014	SC2014	YC2014	ZC2014
FZ2012	0.982						FZ2013	0.486						FZ2014	0.503					
GG2012	0.988	0.974					GG2013	0.669	0.205					GG2014	0.660	0.125				
EC2012	0.990	0.980	0.059				EC2013	0.805	0.431	0.190				EC2014	0.778	0.375	0.191			
SC2012	0.989	0.982	0.127	0.031			SC2013	0.729	0.384	0.410	0.209			SC2014	0.739	0.320	0.372	0.213		
YC2012	0.985	0.981	0.137	0.154	0.016		YC2013	0.676	0.354	0.369	0.379	0.067		YC2014	0.656	0.395	0.391	0.446	0.132	
ZC2012	0.986	0.980	0.503	0.156	0.375	0.084	ZC2013	0.620	0.345	0.304	0.295	0.196	0.053	ZC2014	0.749	0.421	0.398	0.285	0.422	0.074
*	FZ2015	GG2015	EC2015	SC2015	YC2015	ZC2015	*	FZ2016	GG2016	EC2016	SC2016	YC2016	ZC2016	* FZ:MCI GG:II EC:PCOVSI SC:PCOVTI YC:PCOVPI ZC:AFE						
FZ2015	0.406						FZ2016	0.433												
GG2015	0.590	0.192					GG2016	0.662	0.140											
EC2015	0.727	0.428	0.289				EC2016	0.619	0.325	0.139										
SC2015	0.689	0.382	0.367	0.243			SC2016	0.704	0.397	0.274	0.232									
YC2015	0.572	0.354	0.388	0.389	0.094		YC2016	0.574	0.415	0.297	0.341	0.083								
ZC2015	0.550	0.515	0.508	0.299	0.353	0.100	ZC2016	0.554	0.419	0.231	0.298	0.355	0.021							
